# The Relationship between Differentiation of Self and Psychological Adjustment to Separation

**DOI:** 10.3390/healthcare9060738

**Published:** 2021-06-16

**Authors:** Manuel Alfredo Moral, Carlos Alexis Chimpén-López, T. Richelle Lyon, José Carmelo Adsuar

**Affiliations:** 1 Department of Religion, School of Religion, Oakwood University, Huntsville, AL 35896, USA; manuelalfredomoral@gmail.com; 2 Faculty of Nursing and Occupational Therapy, University of Extremadura, Av. de la Universidad, s/n, 10003 Cáceres, Spain; 3 Lyon Consultant, Kerrville, TX 78028, USA; dr.richellelyon@gmail.com; 4 Faculty of Sport Science, University of Extremadura, Av. de la Universidad, s/n, 10003 Cáceres, Spain; jadssal@unex.es

**Keywords:** Bowen’s family system (BFS), Differentiation of Self Short Form (DSI-SF), Psychological Adjustment to Separation (PAST), counseling, assessment, divorce, separation

## Abstract

Many individuals suffer negative mental health consequences such as anxiety and depression following separation from a romantic partner and/or co-parenting conflict due to divorce. Consequently, treating the psychological aftermath of divorce and partner separation remains a predominant concern for mental health practitioners. According to family systems theory, high interdependence and low differentiation of self are associated with a lessened capacity for managing anxiety or adapting to stressful events since intense emotions may inhibit the ability to cope. To assess the relationship between differentiation of self and psychological adjustment to separation, 84 divorced adults completed an online survey. Multiple regression analysis demonstrated that a model based on fusion with others, I-position, and emotional cutoff was a statistically significant predictor of lonely/negativity. Bivariate correlation analyses confirmed significant linear relationships between fusion with others, lonely/negativity, and co-parenting conflict. No differences between genders were found. There is a continuing need to develop interventions to address the negative consequences of divorce, help reduce emotional suffering, and encourage healthy co-parenting. Individuals struggling with psychological adjustment post-divorce, or those seeking education for managing the psychological effects of divorce and co-parenting, may benefit from counseling strategies that incorporate an assessment of differentiation of self and psychological adjustment to separation.

## 1. Introduction

Despite a steady decrease in the frequency of divorce in the United States, and the lowest divorce rate for the past 40 years [1], down to 37% in 2019 as compared to 41% in 2000 [2], treatment for mental health problems related to the psychological aftermath of divorce remains a predominant concern. This includes addressing the effects of recent changes in the divorce process, such as an increased focus on the challenges related to co-parenting following the dissolution of an intimate parental relationship and educating counselors how to support healthy post-divorce co-parenting [3]. As parents separate, family interconnectedness that was once a source of strength may become a source of stress, resulting in challenges to mental health such as emotional distress, depression, or anxiety for members of a family [4]. Children of divorced parents are more likely to demonstrate poor academic performance and experience both emotional and behavioral adjustment problems compared to children from intact families [5,6].

When a relationship that has produced children ends, and at least one of the former partners is not able to successfully manage the process of separation, there is the potential for co-parenting conflicts and long-lasting repercussions for their children. Based on a recent meta-analysis examining the effects of divorce and parental conflict on children’s adjustment, van Dijk et al. [7] validated the existence of a negative correlation between highly conflictual post-divorce family processes and children’s functioning. Divorce accompanied with prolonged parental conflict frequently results in reduced contact between children and the non-residential parent, leading to susceptibility for difficulties with both parents’ and children’s mental and physical health [8]

Amato and DeBoer [9] found long-standing evidence suggesting that divorce transmits across generations; adult children of divorce are approximately twice more likely to divorce themselves, as compared to adult children from intact families based on a weaker commitment to long-term marriage. Gager et al. [10] found that young children exposed to long-term, high levels of parental conflict are more likely to report ending their own romantic relationships. Adult children who have experienced high interparental conflict during their parent’s divorce are especially vulnerable to developing negative psychological consquences such as “loneliness, chronic stress, attachment avoidance, and attachment anxiety” [11] (p. 91) and a pattern of emotional dysregulation that places them at greater risk for challenges related to “psychological well-being and interpersonal competence” [12] (p. 1). As Damota [13] has reported, divorce is not simply a private concern; it is a social issue with repercussions ranging from major life stress at the individual level to deteriorations in both physical and mental health for all family members in the wake of partner separation. For example, a higher incidence of marriage failure is predictive of depression in adults [14]. In addition, for adults who divorce, generalized anxiety particularly appears to be resistant to treatment when accompanied by emotional distress due to the dissolution of a romantic relationship [15].

The variety of complex emotional and behavioral problems resulting from the disruption of divorce can be understood through a grounded theory such as Bowen’s Family Systems, which describes members of a family unit as both interconnected and interdependent based on varying levels of differentiation of the self (DoS) [16]. Bowen [16] introduced the construct of *differentiation of self* as the cornerstone of the eight primary concepts of family therapy, asserting that people differ in levels and intensity of emotional inter-dependence and may be classified according to their relationships with other family members on a scale from well-differentiated to poorly differentiated with gradations in between.

The notion of the family as a system, and the application of this construct to family therapy, was greatly influenced by Bowen [17,18], who extended previous theories that conceptualized families as either intact or not. Bowen depicted families as a set of individuals, with interconnected parts and dynamic interactions, whereby every member has the opportunity to influence each other in positive or negative ways [19,20]. It is imperative that clinicians recognize the persistence and effects of these bonds, even after the breakup of a household (i.e., family members no longer physically live together), and that disturbances in family relationships may be at least partially attributed to DoS and “the intensity of anxiety” that endure within the family system [21] (p. 386). Family members are individuals, and there is variance in one’s ability to achieve self-differentiation and maturity [22] and to manage stress [23]. However, less differentiation (i.e., lower DoS) is generally related to reactivity within familial relationships, especially when anxiety or conflict is high, such as may be present following divorce, due to the propensity for these individuals to become “fused” with the dominant emotions of the family [21]. Low DoS may result in interferences with cognition and communication due to its association with intense fusion, and heightened sensitivity and reactivity within relationships [16,21]. This supports the proposition that DoS has important clinical implications for mental health professionals who either practice multigenerational family systems therapy or who conceptualize clients in terms of the functioning of the nuclear family. Incorporating DoS into the assessment process and/or considering its influence while formulating clinical interventions is likely beneficial due to the insight it provides into the emotional functioning of individuals within the family system, even when that system is no longer intact.

Bowen’s assumptions relied mostly on theory rather than empirical research [17]; however, DoS has been one of the most tested constructs related to the field of family systems [21]. It is the assertion that each family member has a unique capacity to develop autonomy, independence, and intimacy while remaining interconnected to his or her nuclear and extended family [16]. Moreover, an individual who has achieved a healthy DoS is more likely to differentiate from other family members even while maintaining appropriate ties to the system [21,24]. Clinicians can utilize DoS to help individuals identify emotional systems within their families and levels of inter and independence to maintain or strengthen family bonds without sacrificing individual needs [19].

Four dimensions of self-differentiation have been identified through the research [25,26,27]. Individuals who operate primarily from the I-position (IP) are considered to be acting in reality rather than feelings (i.e., rational rather than merely emotional functioning). Emotional reactivity (ER) is defined as acting out according to feelings. Emotional cutoff (EC) is the propensity to emotionally distance oneself from difficult situations. Finally, Fusion with others (FO) describes emotional enmeshment within a family system [17]. It has been evidenced that these dimensions predict increments of the ability to cope with separation anxiety, with the I-position associated with higher levels of psychological adjustment as compared to the other three [21,28,29]

In terms of marital relationships [19] and divorce and mediation [30], high interdependence is associated with the need for approval along with a low capacity for managing anxiety or adapting to stressful events. In relationships with high interdependence, family relations during and after divorce are often affected by each member’s inability to separate personal thoughts and emotions [24]. Individuals with low DoS feel unworthy of love and respect, which intensifies their experience of insecure attachment and inhibits their ability to cope with the psychological adjustment to separation [31,32].

Higher DoS levels are associated with greater emotional balance post-divorce and reduced conflict between ex-spouses [33], whereas lower DoS levels are linked to increased anxiety and negativity. Well-differentiated individuals tend to suffer less [21] and may embody the type of emotional and intellectual maturity required for a successful divorce [34,35]. Achieving high DoS is ideal and allows for both personal identity and identification with members of the original family system such as ex-spouses and children [18,21], even as divorce tends toward an ever-greater complexity of relationships due to remarriages and the creation of new, bi-nuclear families [36]. Highly self-differentiated individuals are more likely to perceive themselves as worthy of love and respect and to maintain secure attachments to other adults, even during challenging situations such as the psychological adjustment to separation [19].

The level of attachment a spouse has toward the partner who initiated the separation is defined as partner attachment [4,37]. High levels of attachment that persist following partner separation are associated with loneliness, psychological distress [22], and anxiety [38]. Although post-separation anxiety is a prominent condition following divorce, some individuals may intentionally push away family members to reduce exposure to further trauma or as an attempt to exert control over their lives [22], which may result in an emotional cutoff or separation from the family.

Psychological Adjustment to Separation (PAS) is defined as the emotional distress that accompanies significant challenges such as acceptance, adaptation, coping, decision-making, and negotiation; factors that frequently require mediation, especially when children are involved [39]. The fragmented structure that results from couple separation tends to produce disparate outcomes based on the individual members’ levels of self-differentiation [40]. Sweeper and Halford [23] created the *Psychological Adjustment to Separation Test* (PAST) to examine partner separation through the constructs of Lonely Negativity (LN), Former Partner Attachment (FPA), and Co-parenting Conflict (CC) based on the assertion that separation from a former partner, such as that which occurs when married or unmarried couples cease cohabitating, is a stressful life event associated with difficulties in adjustment. The authors hypothesized that after separating, spouses will experience different types of trajectories, defined as varying levels of loneliness, psychological distress, or co-parenting conflict based on the individual’s level of emotional attachment to their former partner [23].

Currently, there is a lack of research on the relationship between factors that influence the successful management of partner separation and co-parenting following divorce. Clinicians with knowledge of the relationship between factors such as DoS and PAS, and the negative effects of a lack of self-differentiation on psychological wellbeing, may be better able to help steer clients toward a positive outcome by helping them increase coping skills. For a positive outcome, these challenges should be resolved throughout the processes of separation and divorce, and even post-divorce [4]. Such studies are also needed due to the proliferation of programs that provide education on reducing conflict associated with divorce and co-parenting [3,41]. Our study addressed this need by examining the influence of DoS on adjustment to partner separation, with the hypothesis that lower levels of DoS would be associated with more psychological adjustment problems and co-parenting conflict following partner separation.

## 2. Material and Method

This non-experimental, quantitative, comparative, and cross-sectional study utilized the *Differentiation of Self Inventory-Short Form* (DSI-SF), which included 4 subscales: IP, ER, EC, and FO [17,25]. Each DoS dimension was measured through a Likert scale ranging from 1 (*not at all characteristic of me*) to 6 (*very characteristic of me*). The IP, or I-position, subscale was designed to measure resiliency, stability of identity, and the capacity for communicating personal beliefs, values, or perspectives regardless of stress [17]. It included items such as *No matter what happens in my life, I know that I’ll never lose my sense of who I am* and *There’s no point in getting upset about things I cannot change* [25]. The ER, or emotional reactivity, subscale assessed the tendency to feel overwhelmed in emotional situations [17] using items such as *At times, I feel as if I’m riding an emotional roller-coaster* and *I’m very sensitive to being hurt by others* [25]. The EC, or emotional cutoff, subscale indicated one’s reliance on shutting off emotions in stressful situations [17] with items such as *I tend to distance myself when people get too close to me* and *When one of my relationships becomes very intense, I feel the urge to run away from it* [25]. The FO, or fusion with others, subscale assessed the propensity for fusing in stressful situations such that the individual was unable to independently express personal beliefs, values, or thoughts apart from their significant other [17]. Representative items from this subscale were *I feel a need for approval from virtually everyone in my life* and *I often agree with others just to appease them* [25].

PAS was assessed through the 26-item *Psychological Adjustment to Separation Test* (PAST) by Sweeper and Halford [23], which assessed emotional wellbeing following spousal separation or divorce. Through a combination of exploratory and confirmatory factor analyses, the authors determined a 3-factor structure for the PAST based on (a) lonely negativity (LN), (b) former partner attachment (FPA), and (c) co-parenting conflict (CPC). The 3 PAST subscales utilized a Likert scale ranging from 1 (*strongly disagree*) to 5 (*strongly agree*). Representative items from the LN subscale included *I feel like my life has less purpose in it now* and *I find it hard to do things without a partner* [23]. FPA subscale items consisted of statements such as *Days that have special meaning for my former partner and I are really difficult,* and *I constantly think about my former partner* [23]. 

Both the DSI-SF and PAST have been well-validated. As a shortened form of the original instrument, the DSI-SF was restricted to 20 self-reported items to minimize participant fatigue and potentially increase completion rates [17]. Overall levels of criterion, construct, and convergent validity, and internal consistency estimated for each subscale (i.e., Cronbach’s alpha coefficients) were considered high [17] (see Table 1). The 26-item PAST, another self-report measure, encompassed constructs related to psychological adjustment following partner separation with a comparative *fit index* of 0.90 [23]. According to the authors, each of the 3 replicable factors showed satisfactory test-retest and internal reliability, and good discriminant and convergent validity [23]. 

### 2.1. Ethics Consideration

Before data collection, approval was received for all procedures associated with this study, for conducting research with human subjects, from Northcentral University’s Institutional Review Board of Northcentral University (IRB) (Re: IRB#11191900025G), in concordance with university policies, and with the Declaration of Helsinki and the Ethics and Clinical Research Committee of Northcentral University (IRB, USA, ref. NCU18/2018). As part of the qualifying process, participants were provided with sufficient information to make informed consent. Per the Ethics Code of the American Psychological Association [42], participants should be informed regarding a study’s purpose, expected duration and procedures, the right to decline or withdraw from a study at any time, limits of confidentiality and any known potential risks, discomfort, adverse effects, or benefits [42].

### 2.2. Participants

The sample was U.S. adults at least 21 years old with at least 1 divorce within the past 5 years. Inclusion criteria did not include children from the marriage, but data were collected on this demographic variable to allow for regression of the PAST CPC subscale. An a priori power analysis for the minimum required sample size, using desired *Confidence Interval* of 95% and fair margin of error of 9%, resulting in a desired minimum sample size of 120 divorced adults, and the survey was closed once *N* = 126 individuals completed the online survey. Despite this, the final sample size achieved was *N* = 84, based on complete and engaged responses. The final sample included *n* = 50 males and *n* = 34 females. A total of 65 out of 84 participants reported having children with their former partner, and 19 did not have children.

### 2.3. Measures

DSI-SF scores were calculated in accordance with the recommended procedure [17] as the average of all items within each subscale. The response set for each item ranged from 1 (*not at all characteristic of me*) to 6 (*very characteristic of me*), where participants were asked to report how much each statement concerning their thoughts and feelings about themselves and their relationships with others was generally true for them. PAST scores were a total of responses from a Likert scale ranging from 1 (*strongly disagree*) to 5 (*strongly agree*), with higher scores representing more adjustment problems. Data from Likert scales were a common and useful method for the assessment of the personal attitudes of survey respondents [43]. Though Likert scales were strictly considered to be an ordinal scale of measurement, the data resulting from them and the distances between scale responses can reasonably be considered as equal and meaningful, such that a 1-unit change from 1 to 2 is roughly equivalent to a 1-unit change from 3 to 4 [44]. Further, it is reasonable to treat data from Likert scales as continuous and numeric predictors with adequately large sample sizes and the acknowledgment that both rank and order are lost [44]. Therefore, scale scores based on the DSI-SF and PAST provided appropriate measures for linear regression. Furthermore, parametric tests such as linear regression, where the underlying distribution was assumed to be normal [45], were appropriate analytic procedures for data resulting from Likert scales without increased risk of Type I error or reduction in power [46].

### 2.4. Procedure

The study was open to the general public through Qualtrics, a vendor that provides a commercial platform for hosting online surveys for consumer marketing and academic purposes. Hosting the online survey on Qualtrics also provided a method for recruiting through a variety of social media platforms and distribution channels such as Twitter and LinkedIn [47,48]. Online surveys have become a standard method for recruiting participants and gathering data; they provide a practical alternative to other methodologies and a recruiting process that may be considered fair [49]. The survey began with qualification questions (age and marital status) and informed consent, as approved by the University’s Institutional Review Board (IRB) of Northcentral University, followed by the 20 DSI-SF items and 26 PAST items. Participants were assured that all responses were anonymous and no identifying information was collected. Raw scores were uploaded from Qualtrics into Excel and the scoring method, including reverse scoring as indicated for items with each instrument, was applied. Calculated scores (i.e., averages) for each DSI-SF subscale (e.g., fusion with others, I-position, emotional cutoff, and emotional reactivity) served as the independent/predictor variables. Calculated scores (i.e., totals) for each PAST subscale (e.g., lonely negativity, former partner attachment, and co-parenting conflict) served as the dependent/outcome variables.

### 2.5. Data Analysis

The research question was whether differentiation of self is related to psychological adjustment to separation. This study adds to prior findings that higher levels of self-differentiation are related to both lower levels and fewer symptoms of stress [16,18]. IBM Statistics SPSS 27.0 was used to perform a series of statistical tests. Bivariate correlation analysis (Pearson’ *r*) was conducted for all DSI-SF and PAST variables (i.e., subscales) to evaluate the strength and direction of the linear relationships. Multiple linear regression (enter method in SPSS IBM Statistics, Version 27.0, Armonk, NY, USA) was then conducted to determine if DSI-SF subscales (e.g., FO, IP, EC, and ER) were significant predictors of the 3 dimensions of PAST (e.g., LN, FPA, and CPC). In addition, a study of the internal consistency of each instrument used in this research was conducted in SPSS. Finally, descriptive statistics were calculated for each subscale, and t-tests were performed to assess differences based on gender. The data for this study met the key assumptions for all analyses (e.g., no significant outliers, linear relationships between the variables, independence of observations, minimal collinearity, and approximately normally distributed residuals [50]).

## 3. Results

### 3.1. Internal Consistency and Reliability

It has been recommended that results of *alpha* <0.70 should be assessed with caution [51]. *Cronbach’s alpha* statistics for this sample indicated a moderate level of internal consistency; all but FO were lower than those reported by Drake [17] (see Table 1). As Salkind [45] has noted, reliability estimates are specific to the sample; lower alpha coefficients may reflect a lack of score variability resulting from homogeneousness on some characteristic instead of the larger variance that might be expected from the general population, especially with a smaller sample size. Sweeper and Halford [23] reported a high level of internal consistency for each PAST subscale based on an average of two samples. The instrument was also tested for item–total correlations for all three factors (both samples) and found to be satisfactory at *r* >0.3. Reliability and internal consistency for this sample varied, with a similar though slightly higher alpha coefficient for FPA, and lower values for LN and CPC (see Table 1).

Summary statistics for all scales are reported in Table 2.

### 3.2. Gender Differences

Summary statistics were calculated for each measure based on gender (see Table 3). 

To confirm if any mean difference existed based on gender, independent sample t-tests were conducted in SPSS to assess the effects of gender (male or female) on each DSI-SF and PAST subscale; no significant effects were found (*p* > 0.05) (see Table 4).

### 3.3. Correlation between DSI and PAST 

The following results were obtained based on bivariate correlation analysis in SPSS 27.0 between DSI-SF and PAST subscales (Table 5). A strong linear relationship was found between FO and PAST LN, *r*(82) = 0.570, *p <* 0.001, and a weak to moderate association was found between FO and PAST CPC, *r*(63) = 0.253, *p* = 0.042. There was no significant relationship between FO and PAST FPA, *r* (82) = 0.213, *p* = 0.052, and none between IP and any PAST subscale (*p* > 0.05). EC had a moderate, positive relationship with PAST LN, *r*(82) = 0.451, *p* < 0.001 and with PAST CPC, *r*(63) = 0.338, *p* <0.006, but was not related to PAST FPA, *r*(82) = 0.206, *p* = 0.060. The only significant relationship for ER was with PAST LN, *r*(82) = 0.452, *p* < 0.001. ER was not related to either PAST FPA, *r*(82) = 0.183, *p* = 0.096 or to PAST CPC, *r*(63) = 0.162, *p* = 0.198.

### 3.4. Regression Analyses for DSI-SF as Predictor of PAST

Multiple linear regression analyses (Table 6, Table 7 and Table 8) were conducted to determine if the four DSI-SF subscales (e.g., FO, IP, EC, and ER) were statistically significant predictors of each PAST subscale (e.g., LN, FPA, and CPC). For all study participants (*N* = 84), a model based on DSI-SF subscales significantly predicted the PAST subscale for Lonely Negativity (LN), *F*(4, 83) = 14.914, *p* < 0.001, *R*^2^ = 0.430. Three of the DSI-SF subscales were significant, FO (*p* = 0.002), IP (*p* = 0.025), and EC (*p* = 0.019). The ER subscale was not a significant predictor of LN (*p* = 0.070). A regression model based on DSI-SF subscales did not significantly predict either the PAST Former Partner Attachment (FPA) subscale, *F*(4, 83) = 1.645, *p* = 0.171, *R*^2^ = 0.077 or the PAST Co-Parenting Conflict (CPC) subscale, *F*(4, 60) = 2.283, *p* = 0.071, *R*^2^ = 0.132.

Based on these findings, an additional multiple regression analysis was conducted in SPSS using the stepwise procedure and DSI-SF subscales as predictors for the PAST LN scale. The stepwise method for regression is an alternative, semi-automated procedure used (a) to determine which predictors among a set produces the best-fitting model (i.e., excludes variables to explain the maximum amount of variance in the dependent variable based on the least number of predictors) and (b) to assess changes in *R*^2^, the coefficient of determination [52]. Variables are evaluated based on values of the *t*-statistics for each estimated coefficient, resulting in a useful subset of predictors based on their order of importance in predicting variability of the criterion [53,54]. Three statistically significant models were generated (*p* < 0.001), all excluding the DSI-SF scale for ER. The final and best-fitting model was significant, *F*(3, 83) = 18.219, *p* < 0.001, *R*^2^ = 0.637 and included FO (*p* < 0.001), EC (*p* = 0.009), and IP (*p* = 0.049). The represents a change in *R^2^* and the amount of variance in PAST explained from DoS from 4.3% to 6.4%. 

## 4. Discussion

This study was based on the construct of DoS derived from Bowen Family Systems Theory [16] and two well-validated instruments for measuring DoS and PAS [17,23]. Together, these instruments provided distinct, reliable, valid, and clearly operationalized definitions of the relevant constructs. It was hypothesized that the varying levels of difficulty experienced following divorce, such as emotional suffering due to lonely negativity, former partner attachment, and co-parenting conflict, may be predicted by DoS. The grounding theory was Bowen’s Family Systems, which depicts family members as both interconnected and interdependent units based on an individual’s DoS [16]. 

DoS is influenced by a variety of related constructs that utilize family system theories to conceptualize the family as an inseparable whole [55]. Healthy families demonstrate interconnections between each member in the system, while the individuals give and receive input from one another [16,17,27]. The marriage or union of the parents is a nucleus around which children gravitate and receive powerful influences that either benefit or defeat their own goals and values [4]. DoS is an essential component of psychological wellbeing and directly related to one’s ability to maintain and promote healthy connections with family members even after a divorce, without sacrificing individual thoughts and emotions [20,21]. It is known to be highly influential on one’s ability to psychologically adjust to separation from a spouse [39], especially when accompanied by the persistent need to maintain a family system due to the ongoing needs of mutual children [36]. DoS is likely to affect the degree to which individuals are able to manage significant psychological challenges [36,39]. Low DoS may result in increased dependence on others [24] and a higher incidence of psychological distress when forced to separate from a partner, either by circumstance or by some necessary choice. 

The construct of DoS includes four dimensions indicating higher/positive or lower/negative degrees of DoS within a family system [21]. Across all bi-variate measures, the strongest relationships (*p* < 0.001) were found between DoS and the PAST dimension of Lonely Negativity, such that 32.5% of the variance in LN was explained by FO, 20.3% by EC, and 20.4% by ER. Although the results of this study found evidence that only one PAST subscale was predicted by DoS, the final best-fitting model based on the stepwise procedure utilized FO, IP, and EC as significant predictors (*p* < 0.05), and accounted for 6.4% of the variance in PAST LN. Of these three predictors, Fusion with Others (FO) contributed most strongly to the model, (*t* = 4.681, *p* < 0.001), followed by Emotional Cutoff (EC), (*t* = 2.684, *p* = 0.009), and I-Position (IP), (*t* = 1.996, *p* = 0.049). FO is a product of low DoS; these individuals tend to experience anxiety and familial dependency [37,56]. This likely explains the strength of its relationship to PAST LN, *r*(82) = 0.570, *p* < 0.001, a measure of the degree of lonely negativity (LN) an individual may experience due to separation from a partner after divorce.

Individuals with high FO tend toward extreme emotional responses and are known to overreact to change [29]. This is on the opposite end of the spectrum of DoS, as indicated by stable self-differentiation and the ability to make logical and sound decisions despite the presence of emotional stressors [16,21]. Intense emotions may result in high levels of fusion within a family system, whereby one member sacrificed independence to submit to another, leading to co-dependency, impairment in judgment, and social anxiety [17,29]. Similarly, the finding of a moderately strong, positive relationship with EC and PAST LN is likely explained by its denotation as weak DoS [17,27]. EC refers to an individual’s pattern of separating from stressful emotions by cutting off a relationship or entirely withdrawing from a relationship as an attempt to preserve the self by sacrificing those stressors [16,17,29]. 

Although the regression model confirmed IP as a significant, positive predictor of PAST LN (*p* = 0.025), the bi-variate correlation was not significant, *r*(82) = 0.200, *p* = 0.068. The findings from this study reveal, at best, a weak positive relationship between IP and LN. Based on Bowen’s theory [16], IP provides the base concept for stable DoS [17,27]. High scores on the dimension of IP indicate the ability to make logical, sound decisions outside of emotional stressors that might otherwise cause bias [16,21]. Emotions are subordinated but not removed; rather, they are applied when relevant and helpful for progress and positive development [29]. Therefore, it is not well understood why higher IP would be associated with higher LN for this sample or why no association was found between the DoS subscales and the PAST FPA or CPC subscales; these relationships should be explored in a future study with a larger sample size to increase statistical power, which may help determine the generalizability of these results. Additional research should also incorporate additional demographic data and populations (i.e., beyond heterosexual, legal marriage) such long-term co-habitations or same-sex marriage with or without children. Finally, interactions between the factors could be addressed in a future study.

Implications from this study and proposed future directions for professional practice are that clinical insight may be gained into clients’ specific needs based on their formative experiences as part of an emotionally connected family system. Bowen’s original theory proposed that high DoS is related to one’s capacity for managing stress and navigating the emotional demands of relationships [17,18], whereas low DoS is associated wtih dependence on others [24]. Therefore, it may be reasonable to assume that low DoS is also correlated with a higher incidence of psychological distress for individuals who separate from a former partner, either by circumstance or by some necessary choice. Consequently, adding an assessment of DoS and PAST could prove to be an effective clinical tool for client conceptualization and treatment planning. More studies are needed to further understand the interaction between these factors and to determine how individual DoS dimensions are related to varying levels of psychological adjustment following separation or divorce. This may help clients increase positive future experiences [57] and decrease the risk of negative consequences thus that healthy adjustment and psychological strength after divorce may be encouraged [4].

## 5. Limitations

This study included the following limitations. Given the non-experimental design, it was not possible to draw specific conclusions about a causal relationship. Even though strong linear relationships between variables may be interpreted as meaningful and instructive [58], no observational study design can determine causality between variables [59]. The target minimum sample size for data analysis (*N* = 84) was below the recommended minimum of (*N* = 120). Small sample sizes are always a potential concern in terms of reducing statistical power and increasing the probability of a Type II error [60]. Replicating this study with a larger sample size (>200) may also positively affect the magnitude obtained for the alpha coefficients, which would increase the generalizability of the results [51].

All respondents were self-selected, and all data were self-reported, leading to some risk of non-response bias or social desirability bias [27]; however, this limitation was unavoidable and perhaps less meaningful for this study given that the personal experience of psychological adjustment and self-differentiation are inherently subjective constructs. Attempts were made to reach a wide audience through invitations across social media, but individuals may have excluded themselves based on concerns such as the emotional nature of the subject matter. In addition, by promoting the survey mostly through social media platforms, segments of the population with no participation in, or knowledge of, social media had less chance of being included. This type of convenience sampling is generally less desirable than a purely random sample but does not necessarily negate the validity of the findings [61]. Finally, since over time, symptoms of maladjustment to divorce tend to decrease [62], the study was restricted to those who had experienced a heterosexual divorce within the past five years to control for this potentially confounding variable [63]. Future studies could extend recruitment to couples divorced from same-sex marriage, and potentially include additional demographic variables as covariates or moderators such as race, education, income, time since separation/divorce, length of marriage, number of children, and details of the number and type of remarriage(s) or re-partnering arrangements that followed the partner separation referenced in the study.

## 6. Conclusions

The current study examined DoS, a critical component of Bowen’s family systems theory, in relation to personal psychological adjustment post-divorce. Divorce, by definition, involves not only the legal dissolution of a marital relationship; it dissolves the bonds between a married couple and typically results in a separation of the partners and a disruption of the family system [64]. Individuals vary in levels of psychological distress following separation, with faster recoveries related to higher levels of DoS [15] and higher incidents of persistent depression, debilitating vulnerability, and high anxiety are related to low DoS [65]. The data from this study provided empirical evidence supporting the hypothesis that individuals with the highest levels of Fusion with Others are most likely to be emotionally vulnerable to difficulties in adjusting to separation from their former partner, including problems with lonely negativity and difficulties successfully managing co-parenting conflict. The rupture of a family system is, therefore, a major impetus for the initiation of mental health therapy. Specialized knowledge regarding clients’ levels of DoS and PAS may potentially be utilized to increase both the clinician’s and a client’s insight into the factors that are related to individual reactions and adaptions to emotional distress following separation from a partner, along with the potential to affect families in positive ways as the members separate yet are able to cope with the changes and loss in contact.

Numerous venues for discouraging divorce exist, from religious and secular premarital counseling to post-marital couples counseling, books, and workshops. There is a continuing need to develop specific types of interventions to address the negative consequences of divorce, help reduce emotional suffering, and encourage health co-parenting [3,66]. Clinicians may find that incorporating assessments of DoS and PAST will assist with the processes of client conceptualization and treatment planning for those who initiate mental health counseling due to psychological distress following divorce. Clinicians may wish to consider the impact of DoS in relation to the functioning of family members post-divorce, or as the impetus to probe for details of original family patterns based on the theory of DoS. Clients may also benefit from knowledge about DoS, and its impact on their current relationships. As the association between these factors is better understood, clinicians may gain deeper insight into effective treatments for clients suffering post-divorce through education regarding expected psychological consequences and the need for balance between meeting their own needs (i.e., developing and maintaining self-differentiation) and (especially for those with children) continuing to function within a system that remains somewhat interdependent even as it has broken apart. 

## Figures and Tables

**Table 1:** Comparison of reliability coefficients for measures for the original and current study.

*Ratings (Drake)*			*Current Study*		
DSI-SF Scale/subscale	Items	α	DSI-SF Scale/subscale	Items	α
Fusion with Others	5	0.68	Fusion with Others	5	0.68
I-Position	6	0.70	I-Position	6	0.50
Emotional Cutoff	3	0.79	Emotional Cutoff	3	0.57
Emotional Reactivity	6	0.80	Emotional Reactivity	6	0.70
*Ratings (Sweeper and Halford)*			*Current Study*		
*PAST Scale/subscale*	*Items*	*α*	*PAST Scale/subscale*	*Items*	*α*
Lonely Negativity	11	0.90	Lonely Negativity	11	0.59
Former Partner Attachment	8	0.89	Former Partner Attachment	8	0.92
Coparenting Conflict	7	0.85	Coparenting Conflict	7	0.71

**Table 2:** Summary statistics for differentiation of self-inventory–short form and psychological adjustment to separation test scales.

Variable	Valid	Mean	Median	Mode	St. dev.	Min	Max
Fusion with Others	84	1.86	1.80	1.40	0.55	1.00	3.00
I-Position	84	3.65	3.67	4.00	0.80	1.67	5.17
Emotional Cutoff	84	1.83	1.67	1.67	0.60	1.00	3.00
Emotional Reactivity	84	1.93	1.83	1.67	0.54	1.00	3.00
Lonely Negativity	84	72.95	72.50	72.00	13.23	39.00	97.00
Former Partner Attachment	84	17.87	14.36	8.55	8.91	8.00	36.18
Co-parenting Conflict	65	13.58	14.00	17.00	4.56	7.00	21.00

*Note*. Only participants with children were included in the analysis of co-parenting conflict.

**Table 3:** Descriptive Statistics by Gender.

Scale/Score	Gender	N	Mean	S. Dev.	S.E. Mean
Fusion with Others	Male	50	1.86	0.56	0.08
	Female	34	1.85	0.54	0.09
I-Position	Male	50	3.59	0.72	0.10
	Female	34	3.75	0.91	0.16
Emotional Cutoff	Male	50	1.91	0.55	0.08
	Female	34	1.71	0.64	0.11
Emotional Reactivity	Male	50	1.97	0.55	0.08
	Female	34	1.88	0.54	0.09
Lonely Negativity	Male	50	73.54	12.91	1.83
	Female	34	72.09	13.85	2.38
Former Partner Attachment	Male	50	17.69	9.19	1.30
	Female	34	18.15	8.60	1.47
Co-parenting Conflict	Male	40	13.70	4.45	0.70
	Female	25	13.40	4.82	0.96

**Table 4:** Summary of Gender Differences.

Independent Samples Test									
	Levene’s Test for Equality of Variances		T-Test for Equality of Means						
	*F*	*p*	*t*	df	*p*	Mean Diff	Std. Error Diff	95% Confidence Interval of the Difference	
								Lower	Upper
FO	0.008	0.928	0.058	82	0.954	0.01	0.12	−0.24	0.25
IP	2.211	0.141	−0.898	82	0.372	−0.16	0.18	−0.51	0.19
EC	0.288	0.593	1.581	82	0.118	0.21	0.13	−0.05	0.47
ER	0.065	0.799	0.698	82	0.487	0.08	0.12	−0.16	0.32
LN	0.060	0.808	0.491	82	0.625	1.45	2.96	−4.43	7.33
FPA	0.256	0.614	−0.232	82	0.817	−0.46	1.99	−4.42	3.50
CPC	0.280	0.599	0.256	63	0.799	0.30	1.17	−2.04	2.64

*Note*. Only participants with children were included in the analysis of co-parenting conflict.

**Table 5:** Summary of significance levels for differentiation of self-inventory-short form and psychological adjustment to separation test correlations (*N* = 84).

	Fusion with Others	I-Position	Emotional Cutoff	Emotional Reactivity	PAST LN	PAST FPA
Fusion with Others	---					
I-Position	0.061	---				
Emotional Cutoff	** 0.433	0.001	---			
Emotional Reactivity	** 0.556	−0.095	** 0.360	---		
PAST LN	** 0.570	0.200	** 0.451	** 0.452	---	
PAST FPA	0.213	−0.109	0.206	0.183	** 0.668	---
PAST CPC ***	* 0.253	0.076	** 0.338	0.162	** 0.424	* 0.269

*Note*. Only participants with children included in analysis of co-parenting conflict. * *p* < 0.05. ** *p* < 0.01. *** *N* = 65.

**Table 6:** Differentiation of self-inventory-short form as a predictor for psychological adjustment to separation test lonely negativity subscale.

		95% CI for *B*					
	*B*	*LL*	*UL*	*SE B*	β	*p*	*R_adj_* ^2^
Model						<0.001	0.401
Constant	26.85	12.48	41.22	7.22		0.000	
Fusion with Others	8.52	3.31	13.74	2.62	0.352	0.002	
I-Position	3.25	0.42	6.08	1.42	0.197	0.025	
Emotional Cutoff	5.09	0.87	9.31	2.12	0.229	0.019	
Emotional Reactivity	4.70	−0.39	9.80	2.56	0.192	0.070	

*Note*. Dependent Variable: PAST Lonely Negativity Subscale. *B* = unstandardized regression coefficient; CI = confidence interval; *LL* = lower limit; *UL* = upper limit; *SE B* = standard error of the coefficient; β = standardized coefficient; *R_adj_*^2^ = adjusted R^2^.

**Table 7:** Differentiation of self-inventory-short form as a predictor for psychological adjustment to separation test former partner attachment subscale.

		95% CI for *B*					
	*B*	*LL*	*UL*	*SE B*	β	*p*	*R_adj_* ^2^
Model						0.171	0.030
Constant	13.21	0.90	25.52	6.18	0.14	0.036	
Fusion with Others	2.22	−2.24	6.69	2.24	−0.11	0.325	
I-Position	−1.25	−3.68	1.18	1.22	0.13	0.308	
Emotional Cutoff	1.93	−1.69	5.54	1.82	0.05	0.291	
Emotional Reactivity	0.82	−3.55	5.18	2.19	0.19	0.711	

*Note*. Dependent Variable: PAST Former Partner Attachment Subscale. *B* = unstandardized regression coefficient; CI = confidence interval; *LL* = lower limit; *UL* = upper limit; *SE B* = standard error of the coefficient; β = standardized coefficient; *R_adj_*^2^ = adjusted R^2^.

**Table 8:** Differentiation of self-inventory-short form as a predictor for psychological adjustment to separation test co-parenting conflict subscale.

		95% CI for *B*					
	*B*	*LL*	*UL*	*SE B*	β	*p*	*R_adj_* ^2^
Model						0.171	0.074
Constant	6.39	−0.65	13.43	3.52	0.13	0.075	
Fusion with Others	1.03	−1.45	3.52	1.24	0.06	0.408	
I-Position	0.34	−1.01	1.70	0.68	0.28	0.616	
Emotional Cutoff	2.07	0.08	4.06	1.00	0.01	0.042	
Emotional Reactivity	0.07	−2.46	2.60	1.27	0.192	0.956	

*Note*. Only participants with children included in analysis of co-parenting conflict. Dependent Variable: PAST Co-Parenting Conflict Subscale. *B* = unstandardized regression coefficient; CI = confidence interval; *LL* = lower limit; *UL* = upper limit; *SE B* = standard error of the coefficient; β = standardized coefficient; *R_adj_*^2^ = adjusted R^2^.

## Data Availability

Not applicable.
